# The evaluation of metallothionein expression in nasal polyps with respect to immune cell presence and activity

**DOI:** 10.1186/1471-2172-11-10

**Published:** 2010-03-09

**Authors:** Magdalena Dutsch-Wicherek, Romana Tomaszewska, Agata Lazar, Paweł Stręk, Łukasz Wicherek, Krzysztof Piekutowski, Wojciech Jóźwicki

**Affiliations:** 1 Otolaryngology Head and Neck Surgery Department, Jagiellonian University, Sniadeckich 2, Krakow 31-531, Poland; 2 Pathomorphology Department, Jagiellonian University, Grzegorzecka, 31-530 Krakow, Poland; 3 Gynecology and Oncology Department of the Lukaszczyk Oncological Center in Bydgoszcz and Chair of Gynecology, Oncology and Gynecological Nursing of the Ludwik Rydygier Medical College in Bydgoszcz, Mikolaj Kopernik University, Poland; 4 Department of Tumor Pathology and Pathomorphology the L. Rydygier Collegium Medicum UMK F. Lukaszczyk Oncology Center, Bydgoszcz Poland

## Abstract

**Background:**

The expression of metallothionein (MT) is involved in acquiring resistance to immune-mediated apoptosis; it is also a negative regulator of the immune response. Nasal polyps are typified by a resistance to immune-mediated apoptosis as well as by excessive immune cell infiltration. RCAS1 (receptor-binding cancer antigen expressed on SiSo cells) is a membrane protein capable of inducing the apoptosis of CTLs and NK cells. The aim of the present study has been to explore the expression of metallothionein with respect to immune cell presence and immune cell activity. In our study, we identified immune cells using CD4 and CD68 antigen expression and evaluated their activity using CD25 antigen expression. We then analyzed metallothionein, RCAS1, CD25, CD4, and CD68 in a sampling of 50 nasal polyps using the immunohistochemistry method. We were able to divide the nasal polyps into three main groups according to their predominant immune cell infiltration: eosinophilic nasal polyps (21 cases), lymphocytic nasal polyps (17 cases), and neutrophilic nasal polyps (12 cases).

**Results:**

In the present study, statistically significant differences between the MT expression in the epithelium and that in the stroma of the nasal polyps along with the accompanying alterations in activation markers on immune cells were found and the number of macrophages in both the eosinophilic and the lymphocytic nasal polyps was assessed. RCAS1-expressing macrophages were found only in the eosinophilic nasal polyps.

**Conclusion:**

MT expression seems to favor the survival of nasal polyp epithelial cells in the adjacent area of increasingly cytotoxic immune activity. RCAS1-expressing macrophages seem to participate in creating the immune suppressive microenvironment and so help to sustain local inflammation.

## Background

Chronic rhinosinusitis with nasal polyps is considered a subdisease of chronic rhinosinusitis [1]. Nasal polyps are found in 20% of the cases of chronic rhinosinusitis [2], and most polyps originate in the clefts of the osteomeatal complex. The overall prevalence rate for the disease in the general population ranges from 1-4% [3,4]. The immune cell infiltration identified in nasal polyps is a mixed one; it includes eosinophils, and these constitute more than 10% of the cells of nasal polyps in the Caucasian population. The number of CD3 positive and CD25 positive activated T lymphocytes also increases in nasal polyps [5]. The pattern of immune cell infiltration to the polyps in non-allergic as opposed to allergic patients who have chronic rhinosinusitis with nasal polyps has been demonstrated to be different. In non-allergic patients, fewer CD4+ cells in the epithelium and more CD8+ cells in the lamina propria were found than in the same tissues of the patients who had allergies. The number of macrophages increases as well in nasal polyps. These cells have enhanced mannose receptor expression, which is capable of phagocytosis and signals transduction for pro-inflammatory mechanisms [6,7].

The histomorphological characterization of nasal polyp tissue reveals frequent epithelial damage, a thickened basement membrane, and edematous or sometimes fibrotic stromal tissue with a reduced number of vessels and glands [3].

Nasal polyps constitute a multifactorial disease. Significant participating factors include infectious as well as noninfectious inflammation and anatomic and genetic abnormalities. Allergic and nonallergic rhinitis as well as allergic fungal sinusitis, aspirin intolerance, asthma, cystic fibrosis, primary ciliary dyskinesia, and Kartagener syndrome are all associated with nasal polyps. Furthermore, nasal polyps are believed to represent the final stage of chronic nasal inflammation [1].

As in our previous study, we demonstrated that nasal polyps develop resistance to immune-mediated apoptosis and are able to express factors by which the activity of infiltrating immune cells can be regulated. In the present study we have focused on the analysis of metallothionein in nasal polyps [8].

Metallothionein (MT) is a small (approx. 7 kDa), thiol-rich protein [9]. The expression and induction of metallothionein has been associated with protection against both oxidative stress and apoptosis. This is because thiols participate in complexing with divalent metal cations. When metallothionein binds to essential divalent metals (zinc and copper), it may serve as a metal reservoir for apo-enzymes and zinc-finger transcription regulators [10]. Apoptosis has also been increased in MT-null cells [11]. MT has been found to be associated with the enhanced proliferation of cells in esophageal, breast, and nasopharyngeal cancers [12-14]. Since MT function includes the maintenance of proper intracellular Zn^2+ ^level, and the proper intracellular Zn^2+ ^level is responsible for the regulation of caspase-3 activity, Zn^2+ ^has been observed to inhibit caspase-3 activity. Caspase -3 controls the essential step of apoptosis and is responsible for DNA fragmentation [15]. The potential role of metallothionein in the modulation of apoptosis may depend on the nuclear/cytoplasmic localization of MT in the cell. Cytoplasmic MT is thought to protect against cytotoxicity whereas nuclear MT protects against genotoxicity [16].

E. Canpolat *et al. *has proposed that MT may serve as a negative regulator of the immune response and so suppress the autoimmune attack on self-tissues. Indeed, the induction of MT by glucocorticoids may explain the role they play in treating autoimmune diseases [17].

The aim of the present study has been to evaluate MT immunoreactivity in nasal polyps with respect to the type of immune cell infiltration present. For this purpose, we have considered the presence of macrophages, CD4 positive Th lymphocytes, and CD25 antigen immunoreactivity. We have also repeated the analysis of RCAS1 expression so that we can consider its expression in relation to immune cell presence and activity. The major function of RCAS1 expression is to inhibit activated immune cells, such as T and B lymphocytes and NK cells, and to induce their apoptosis. RCAS1 as expressed on cancer cells is responsible for both tumor escape from host immunological surveillance and the creation of immune tolerance toward tumor cells [17,18]. Moreover, it has been shown that macrophages stimulated with LPS (lipopolysaccharide)-enhanced RCAS1 expression together with the induced apoptosis of the progenitor cells of the erythroblast line through RCAS1 play an important regulatory role in erythropoesis [19,20]. Furthermore, it has been shown that RCAS1 can be found only on activated monocytes [19,21]. Because RCAS1-positive macrophages represent a population of cells capable of regulating immune system cell activity in a negative manner, their presence in the nasal polyp microenvironment may also help to create a local immunosuppressive microenvironment.

## Results

### The analysis of MT expression in nasal polyps

Metallothionein immunoreactivity was identified in all the examined nasal polyp tissue samples and this immunoreactivity was intracellular.

#### Eosinophilic nasal polyps

MT immunoreactivity was found in all the examined tissue samples derived from the eosinophilic nasal polyps and was present in the epithelium lining the nasal polyps; it represented a nuclear pattern of staining (Table 1). MT immunoreactivity was also present in the stroma of nasal polyps where it represented a nuclear and cytoplasmic staining pattern (Table 2).

**Table 1:** MT immunoreactivity in the epithelium of nasal polyps.

Variables	Number	MT immunoreactivity in the epithelium			
		
		-	+1	+2	+3
Eosinophilic nasal polyps	21	0	2 (10%)	19 (90%)	0
Lymphocytic nasal polyps	17	0	4 (24%)	13 (76%)	0
Neutrophilic nasal polyps	12	0	12 (100%)	0	0

**Table 2:** MT immunoreactivity in the stroma of nasal polyps.

Variables	Number	MT immunoreactivity in the stroma			
		
		-	+1	+2	+3
Eosinophilic nasal polyps	21	0	4 (19%)	15 (71%)	2 (10%)
Lymphocytic nasal polyps	17	0	7 (42%)	9 (53%)	1 (5%)
Neutrophilic nasal polyps	12	0	12 (100%)	0	0

**Figure 1:**
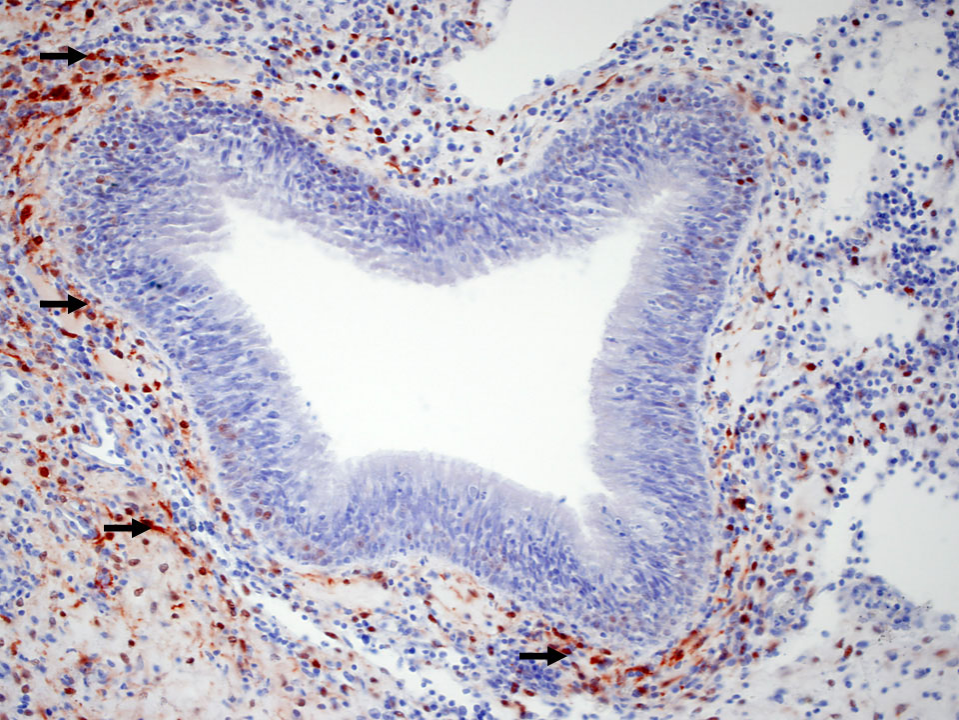
**MT immunoreactivity in the stroma of nasal polyps (arrows)**.

Figure 1. MT immunoreactivity in the stroma of nasal polyps.

#### Lymphocytic nasal polyps

MT immunoreactivity was identified in all the examined lymphocytic nasal polyps, and as was the case with the eosinophilic nasal polyps, was found to be present in both the epithelial cells and the stroma (Table 1, Table 2).

Table 1. MT immunoreactivity in the epithelium of nasal polyps.

Table 2. MT immunoreactivity in the stroma of nasal polyps.

**Figure 2:**
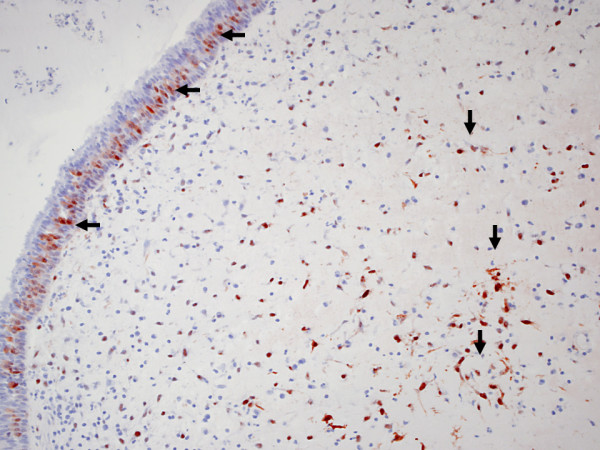
**MT immunoreactivity in the epithelium (horizontal arrows) and stroma (vertical arrows) of nasal polyps**.

Figure 2. MT immunoreactivity in epithelium (horizontal arrows) and stroma (vertical arrows) of nasal polyps.

#### Neutrophilic nasal polyps

The MT staining pattern was weak in the epithelium lining both the polyps and the stroma. No other examined antigens (such as CD25, CD4, or CD68) were identified in the neutrophilic nasal polyps. On account of these findings, the neutrophilic nasal polyps were excluded from further consideration in the study.

Interestingly, exceptionally high MT expression was found in the stroma of the nasal polyps that were adjacent to bone. Moreover, the osteoblasts of the adjacent bone showed high levels of MT immunoreactivity. This immunoreactivity was observed in all the bone fragments obtained for the study with nasal polyps regardless of the type of the immune cell infiltration in the polyps. No other bone fragment regions exhibited MT immunoreactivity.

**Figure 3:**
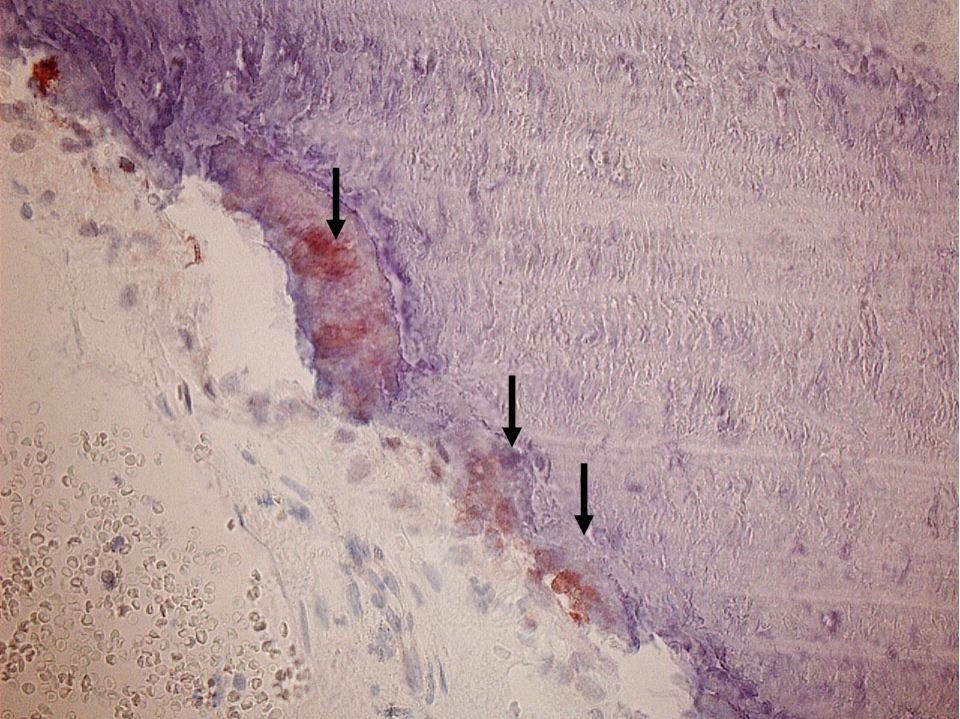
**MT immunoreactivity in the bone adjacent to nasal polyps (arrows)**.

Figure 3. MT immunoreactivity in the bone adjacent to the nasal polyps.

#### Comparison of MT immunoreactivity in eosinophilic and lymphocytic nasal polyps

MT expression within the epithelium was statistically significantly higher in the eosinophilic than in the lymphocytic nasal polyps (p = 0.02). MT stromal expression was higher in the eosinophilic nasal polyps than in the lymphocytic, but the difference was not statistically significant. A statistically significant correlation, however, was found between MT expression in the epithelium and in the stroma (r = 0.61, p < 0.0001).

### The analysis of immune cells in nasal polyps

#### CD68 positive cells

CD 68 positive cells (macrophages) were identified in both the stroma and the epithelium lining the nasal polyps. The number of scattered cells in the stroma ranged from 0 to 3 in a hpf. In the edematic area of the nasal polyp stroma, the number of inflammatory cells was high, and the number of CD68 positive cells increased to 8-11 in a hpf. Overall, the number of macrophages was statistically significantly higher in the tissue of the eosinophilic nasal polyps than in that of the lymphocytic nasal polyps (p < 0.01) (Table 3).

**Table 3:** The immunoreactivity of CD68 in nasal polyps.

Variables	Number	CD68 immunoreactivity in nasal polyps			
		
		-	+1	+2	+3
Eosinophilic nasal polyps	21	0	0	1 (4.8%)	20 (95.2)
Lymphocytic nasal polyps	17	0	0	7 (41.2%)	10 (58.8%)

Table 3. CD68 immunoreactivity in nasal polyps.

### The analysis of RCAS1

While in our previous study we identified RCAS1 expression in nasal polyps, in the current study we have also evaluated this expression in nasal polyps [8]. Strong cytoplasmic RCAS1 immunoreactivity was found in the epithelium lining the polyps, and was additionally present in the extra-cellular mucus. Moreover, RCAS1 immunoreactivity was observed in the mucous glands, but was weaker than in the surface epithelium. As in the previous study, the RCAS1 immunoreactivity level in the epithelium lining the polyps was significantly higher in the lymphocytic nasal polyps than in the eosinophilic polyps. Likewise, in the present study the level of RCAS1 immunoreactivity in the epithelium lining the nasal polyps was significantly higher in the lymphocytic nasal polyps than in the eosinophilic polyps (p < 0.05) (Table 4). Additionally the stroma of eosinophilic polyps contained single RCAS1 positive cells that were dispersed within the immunological infiltration and were identified as CD68 positive cells. These same cells were not found in the lymphocytic nasal polyps.

**Table 4:** The immunoreactivity of RCAS1 in nasal polyps

Variables	Number	RCAS1 immunoreactivity in nasal polyps			
		
		-	+1	+2	+3
Eosinophilic nasal polyps	21	0	19 (90%)	2 (10%)	0
Lymphocytic nasal polyps	17	0	10 (58.8%)	4 (23.5%)	3 (17.7%)

Table 4. RCAS1 immunoreactivity in the epithelium of nasal polyps.

**Figure 4:**
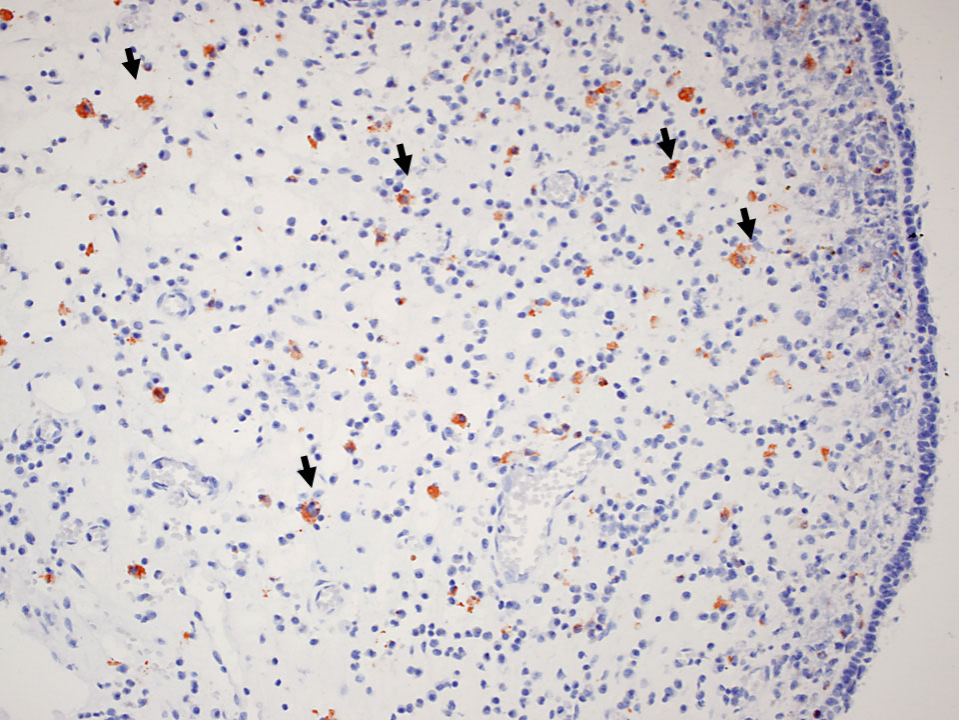
**RCAS1 positive macrophages within eosinophilic nasal polyps (arrows)**.

Figure 4. RCAS1 positive macrophages within eosinophilic nasal polyps.

CD68 RCAS1 positive cells were observed to migrate from the stroma of the nasal polyps through the epithelium (single cells seen within the epithelium) to the mucus.

**Figure 5:**
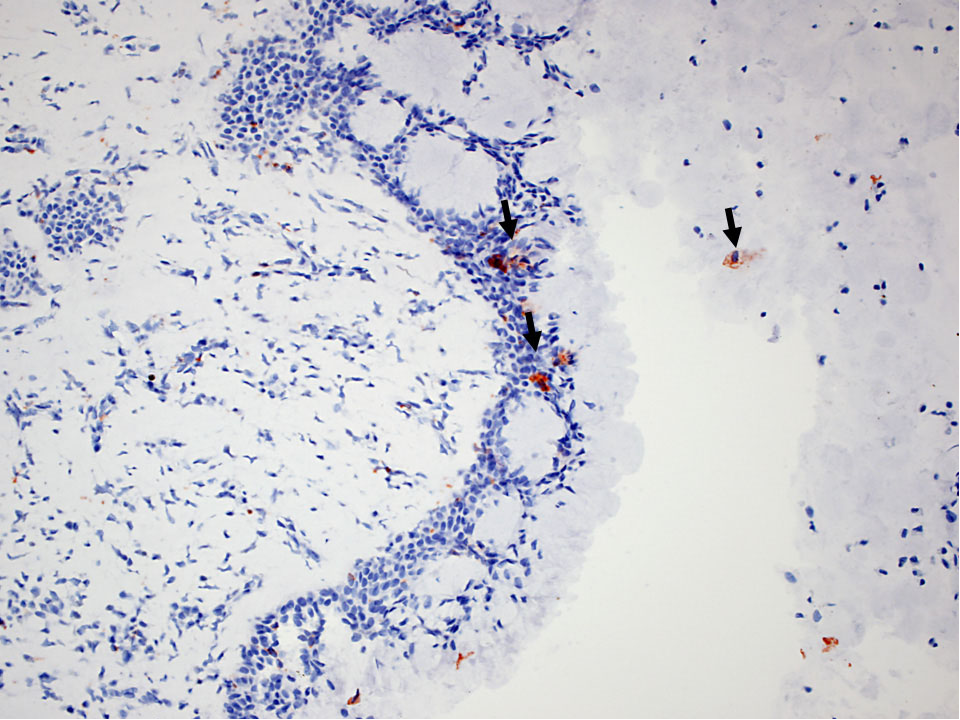
**RCAS1 positive macrophages migrating through the epithelium of nasal polyps to the mucus (arrows)**.

Figure 5. RCAS1 positive macrophages migrating through epithelium of the nasal polyps to the mucus.

CD4 antigen expression was observed in 47.65% of the eosinophilic nasal polyps and in 66.7% of the lymphocytic nasal polyps. No differences, however, were observed between the eosinophilic and lymphocytic nasal polyps with respect to CD4 expression (Table 5).

**Table 5:** The immunoreactivity of CD4 in nasal polyps.

Variables	Number	CD4 immunoreactivity in nasal polyps			
		
		-	+1	+2	+3
Eosinophilic nasal polyps	21	11 (52%)	0	8 (38%)	2 (10%)
Lymphocytic nasal polyps	17	5 (29)	0	7 (42%)	5 (29)

Table 5. CD4 antigen immunoreactivity in nasal polyps.

CD 25 expression was statistically significantly higher in the eosinophilic nasal polyps than in the lymphocytic nasal polyps (p = 0.03) (Table 6).

**Table 6:** The immunoreactivity of CD25 in nasal polyps

Variables	Number	CD25 immunoreactivity in nasal polyps			
		
		-	+1	+2	+3
Eosinophilic nasal polyps	21	2 (9.5%)	0	5 (23.8%)	14 (66.7%)
Lymphocytic nasal polyps	17	0	0	10 (58.8%)	7 (41.2%)

Table 6. CD25 immunoreactivity in nasal polyps.

### The comparative analysis of MT and RCAS1 expression and immune cell activity

A statistically significant correlation was found between CD25 expression on immune cells and MT expression in the epithelium of nasal polyps (r = 0.37, p = 0.02) as well as between CD25 expression on immune cells and MT expression in the stroma of the nasal polyps (r = 0.35, p = 0.03). No statistically significant correlations were found, however, between MT and RCAS1 immunoreactivity on these same cells and tissues.

## Discussion

In the present study, statistically significant differences in MT expression in both the epithelium and stroma of the nasal polyps with accompanying alterations in activation markers on immune cells and in the number of macrophages were found when comparing the eosinophilic and lymphocytic nasal polyps. In our previous study, we demonstrated that nasal polyp tissue develops a resistance to immune-mediated apoptosis in accordance with increasing immune cell infiltration. This has been recognized by evaluating the expression of DFF-45 and depended on the predominant immune cell infiltration profile. Nasal polyp tissue has also been shown to regulate the activity of infiltrating immune cells by the expression of RCAS1, a protein that inhibits the activity of immune cells and induces their apoptosis [8]. To our knowledge, this is the first investigation concerning MT immunoreactivity in nasal polyps with regard to the type of immune cell infiltration.

Nasal polyps are infiltrated by a variety of immunological cells---eosinophils, neutrophils, plasma cells, mast cells, and macrophages, lymphocytes, and their subgroups have all been found in nasal polyps [22]. The lymphocytes that infiltrate nasal polyps have been identified as predominantly memory T cells in an activated state and these produced a mixed Th1/Th2 cytokine pattern (IFN-gamma and IL-5) [23]. In our study, no differences were observed between the lymphocytic and eosinophilic nasal polyps as far as CD4 antigen expression. The flow cytometry analysis of lymphocytes isolated from nasal polyps revealed a significant increase of IL-2 receptor and ICAM-1 molecule expression on T cells isolated from nasal polyps when compared with peripheral blood lymphocytes [24]. In our study, CD25 expression was present in both the eosinophilic and lymphocytic nasal polyps and the level of expression was statistically significantly higher in the eosinophilic nasal polyps than in the lymphocytic polyps. This may be a result of the intensity of the immune response in these polyps and may suggest a greater number of cells able to respond to the increased IL-2 expression. Higher CD25 immunoreactivity in the eosinophilic nasal polyp stroma in comparison to that in lymphocytic nasal polyp stroma may also suggest the presence of Treg cells typified by CD25 expression and may result from the level of immune tolerance developing in the polyp microenvironment due to chronic inflammation. Additionally, in the present study, a statistically significant correlation was found between CD25 and MT immunoreactivity in the stroma of the nasal polyps (r = 0.35, p = 0.03). While on the one hand this correlation may result from MT immunoregulatory function [16], on the other hand it could be the result of the development of the resistance to immune-mediated apoptosis in the stromal cells that are exposed to excessive immune response in the microenvironment of the nasal polyps and so may constitute a protective reaction by these cells [16,25].

The accumulation of mannose-receptor positive macrophages that has been reported in nasal polyps in cell aggregates suggests that these cells play a key role in the pathogen-macrophage interaction in nasal polyps [7]. The number of macrophages in our study was statistically significantly higher in the eosinophilic than in the lymphocytic nasal polyps. RCAS1-positive macrophages were identified only in the eosinophilic nasal polyps. This may be due to the different type of immune infiltration pattern in these kinds of nasal polyps. RCAS1-positive macrophages are exclusively identified in normal and pathological conditions; moreover, they have been identified immunohistochemically among macrophages in hematopoietic tissue [19]. Additionally, RCAS1-positive macrophages have been found in the peripheral blood of patients with Hodgkin's lymphoma, those with inflammatory liver diseases, and those suffering from ovarian endometriosis [21,20,26,27]. Because RCAS1 is responsible for the regulation of immune cell activity and may induce immune cell apoptosis, it is possible that it participates in the immune suppression phenomenon [28,29]. RCAS1-positive macrophages may indeed suppress the activity of the infiltrating immune cells. This mechanism may in turn help to develop immune tolerance so that the nasal polyps maintain their growth and development. Matushima *et al. *observed RCAS1 only on activated macrophages in bone marrow [19]; Enjoji *et al.*, however, observed RCAS1-expressing macrophages in the livers of patients with inflammatory liver diseases [21]. Furthermore, the number of macrophages increased as the inflammation increased [21]. In general, two types of macrophages can be identified according to their respective functions: one type promoting an antitumor immune response and the second type promoting a Th2 response in the tumor microenvironment. The M1 phenotypes interleukin 12 high, interleukin 23 high, and interleukin 10 low, along with the production of reactive oxygen and nitrogen intermediates and inflammatory cytokines, induce effector cells in Th1 responses. By contrast, the M2 phenotypes, interleukin 12 low, interleukin 23 low, and interleukin 10 high, participate in polarized Th2 responses. M2 tumor-associated macrophages were shown to promote tumor proliferation and progression, stimulate angiogenesis, and inhibit adaptive immunity [30]. Since RCAS1-positive macrophages seem to exhibit a regulatory function, we speculate that they belong to the M2 phenotype, and we have recently described their presence in the head and neck cancer microenvironment [31].

The presence of RCAS1-positive macrophages in eosinophilic nasal polyps may only prove that lymphocytic nasal polyps differ from eosinophilic polyps with respect to the degree of the disturbance in immune response regulation that can be detected in the nasal polyp microenvironment. The ongoing inflammation in the nasal polyps may lead to a change in the function of the macrophages related possibly to RCAS1 expression and leading to the creation of the immune suppressive microenvironment, thus helping to sustain local inflammation. This may affect both the course of the disease and the outcome of the treatment. Furthermore, such polyps tend to recur.

The local inflammation enhances the infiltration of immune cells and the accumulation of inflammatory mediators that may injure the adjacent epithelium, thus inducing the apoptosis of healthy cells. Normal cells acquire resistance to immune-mediated apoptosis in order to protect themselves against the inflammatory process. The increasing resistance to immune-mediated apoptosis was identified in our previous report by the presence of decreased DFF-45 protein expression in nasal polyp tissue [8] and most likely reinforces the development of nasal polyps. MT was established as an anti-apoptotic protein [32]. Intracellular MT expression in nasal polyps may assure that a resistance to immune-mediated apoptosis in nasal polyps is acquired. On the one hand, MT seems to play a protective role against apoptosis chiefly by maintaining the proper level of intracellular zinc ions. This is because the level of these intercellular ions is directly responsible for caspase-3 activity, which is in turn responsible for the DNA cleavage [33]. On the other hand, MT may also play an important immunomodulating role and some have proposed that it is a negative regulator of immune response [16]. The level of MT expression in our study depended on the pattern of immune infiltration and was found in both the stroma tissue and bone fragments adjacent to the nasal polyps, indicating the reaction of the entire adjacent tissue to the increasing aggressiveness of the immune response.

It has been demonstrated that the spread of the inflammation in chronic rhinosinusitis may be related to the inflammation taking place within the bony walls underneath the inflamed mucosa of chronic rhinosinusitis. The histopathological examination of ethmoid bone fragments obtained from patients with chronic rhinosinusitis revealed the presence of bone remodeling along with the features of neo-osteogenesis [34]. „Osteitis” is defined as a superficial inflammation of the bones without the bone marrow spaces (flat bones) and is a variant of osteomyelitis [35]. Because chronic rhinosinusitis may induce the inflammatory process in the bones of the paranasal sinuses, the presence of MT immunoreactivity in the bones adjacent to inflamed mucosa may also be a manifestation of the spread of the inflammatory process.

## Conclusions

MT expression seems to favor the survival of nasal polyp epithelial cells in the adjacent area of increasingly cytotoxic immune activity. Additionally, the RCAS1- positive macrophages present only in the eosinophilic nasal polyps seem to be involved in creating the suppressive microenvironment in nasal polyps.

## Methods

### Clinical material

Tissue samples were derived from the ENT Head and Neck Surgery Department of the Jagiellonian University during routine endonasal sinus surgery. The patient's consent was obtained in each case. Additionally, approval for the research program was granted from the Ethical Committee of the Jagiellonian University in Krakow: KBET/90/B/2005. We recruited 100 patients from those who had undergone functional endoscopic sinus surgery between January 2005 and November 2006. From this group of patients, 50 were selected for our study. All the tissue samples were histopathologically verified. Pathological analysis using the classical hematoxylin and eosin staining techniques after fixation in formalin of the surgically removed material was performed in the Pathology Department of the Jagiellonian University by an experienced pathologist. The predominant immune cell infiltration in the nasal polyps was determined under histopathological examination. Based on this examination, three distinct types of polyps were selected---eosinophilic nasal polyps, lymphocytic nasal polyps, and neutrophilic nasal polyps. The criteria for the selection of the different types of nasal polyps were as follows:

Lymphocytic nasal polyps had a predominant infiltration of mononuclear cells; the percentage of eosinophilic cells in these polyps did not exceed 10%, and the basal membrane should not be thickened. Eosinophilic nasal polyps had a predominantly eosinophilic cell infiltration (not less than 90%), and the basal membrane was thickened. Polyps that did not fulfill the above criteria, but were infiltrated mainly by eosinophils with the percentage of these cells exceeding 50%, were classified as eosinophilic. Lastly, neutrophilic nasal polyps were infiltrated mainly by neutrophils.

The tissue samples were divided into three main groups according to the predominant immune cell infiltration: nasal polyps predominantly infiltrated by eosinophils or eosinophilic nasal polyps (21 cases); nasal polyps predominantly infiltrated by lymphocytes or lymphocytic nasal polyps (17 cases), and nasal polyps predominantly infiltrated by neutrophils or neutrophilic nasal polyps (12 cases).

The clinical characteristics of the subjects are presented in Table 7.

**Table 7:** The clinical characteristics of the patient group.

Clinicopathological findings	Nasal polyps (n = 50)		
	
	Lymphocytic (n = 17)	Neutrophilic (n = 12)	Eosinophilic (n = 21)
Mean age	47 (± 14.96)	51 (± 16.9)	45.9 (± 15.67)

Male	64.7 (11)	50 (6)	52.4 (11)
Female	35.3 (6)	50 (6)	47.6 (10)

Microbial bacterial infections *Staphylococcus aureus* *Streptococcus* *Enterococcus* *Echerichia coli*	-	41.6 (5)	23 (3)

Microbial fungal infections *Aspergillus*	-	-	19 (4)

Asthma	12 (2)	-	38 (8)

NSAID intolerance	-	-	42 (9)

Documented allergy	23 (4)	-	61 (13)

Nasal septum deviation	12 (2)	-	10 (2)

All figures refer to the percentage of samples; n is the number of patients

In the group of patients classified as suffering from lymphocytic nasal polyps, no concomitant diseases were found though some individual patients did have asthma and there were patients with single documented allergies. The patients with eosinophilic nasal polyps differed significantly from those with lymphocytic nasal polyps.

### Immunohistochemistry

Immunohistochemical analysis was performed in the Pathology Department of the Jagiellonian University. Five-micrometer slides from each case were stained to visualize the expression of RCAS1, MT, and CD4-, CD25-, and CD68-positive cells. In all cases, immunohistochemistry was performed applying the Envision method using Dako Autostainer. The following antibodies were applied: mouse monoclonal antibody Anti- RCAS1 (Medical and Biological Laboratories, Naka-ku Nagoya, Japan in DAKO Antibody Diluent with Background Reducing Components-DAKO, Denmark, dilution 1:1000), monoclonal mouse antibody ImmunOTM (MP Biomedicals, Inc., clone 1A12 in dilution 1:1000), CD68 (DAKO, clone PG-M1 in dilution 1:50), CD4 (Novocastra, clone IF6 in dilution: 1:50), CD25 (Interleukin-2 Receptor, NCL-CD25-305, Novocastra in dilution 1:25), according to the manufacturer's instructions. Visualization of reaction products was performed using AEC (3-amino-9-ethyl-carbazole) as a chromogen (AEC Substrate Chromogen ready-to-use, DAKO, Denmark) for 10 minutes at room temperature. Sections were counterstained with hematoxylin and mounted in glycergel. As a positive control, a tonsil specimen was taken for RCAS1, while for metallothionein a breast cancer specimen was obtained. All the stainings were performed with the same procedure but with the omission of the primary antibody as a negative control. RCAS1 expression was evaluated in entire slides in the area of nasal polyps, the epithelium, and the stroma according to the following scale: 0 - no reactivity; +1 - weak, when observed any (also granular in the paranuclear region) cytoplasmic staining pattern (in up to 10% of positive cells); +2 - marked cytoplasmic (sometimes together with membranous) staining in 11-30% of the cells; +3 - high expression (more than 30% of positive cells). The degree of metallothionein positivity was quantified as the percentage of MT-positive cells in the nasal polyp slides. The staining in the epithelial and stromal cells of the nasal polyps was evaluated according to the following scale: 0 - lack of any positivity; 1+ - weak staining in less than 5% of the cells; 2+ - moderate - various staining intensity but in <50% of the cells, 3+ - strong - staining of more than 50% of the cells. The immune cells were calculated in an entire specimen, in the region of nasal polyps and an average cell number per 1hpf (high power field, objective magnification ×40) was calculated. Variable scales were used to evaluate an amount of cells semi-quantitatively, depending on their general number in the specimen. CD25+, CD4+, and CD68+ cells were estimated according to the following scale: 0 - lack of positive cells; +1 - single positive cells in the specimen; +2 - 1-5 positive cells per 1hpf; +3 - more than 5 positive cells/1hpf.

### Statistical analysis

The distribution of variables in the examined groups of patients checked with the use of the Shapiro-Wilk test showed that all of them were different from normal. Non-parametric testing was therefore employed. The statistically significant difference between the groups was determined by the Kruskal-Wallis analysis of variance (ANOVA) test. The Mann-Whitney U test was then used as appropriate. The data in the Tables is presented as means ± standard error of the mean (SEM). A p value of < 0.05 was accepted as statistically significant. The Spearman Rank Test was used to evaluate interclass correlation coefficients. All calculations were carried out with the use of STATISTICA software v. 6 (StatSoft, USA, 2001).

## Authors' contributions

MDW conceived of the study, designed the study, analyzed and interpreted data, and drafted the manuscript. RT carried out the molecular study and revised the study. AL carried out the molecular study. PS participated in the sequence alignment. LW participated in the final correction of the manuscript. KP participated in the final correction of the manuscript. WJ participated in the final correction of the manuscript. All authors read and approved the final manuscript.
